# Cross-Cultural Adaptation and Pilot Psychometric Validation of the European Organisation for Research and Treatment of Cancer—Quality of Life Questionnaire—Sexual Health (EORTC QLQ-SH22) Scale, Moroccan Arabic Version

**DOI:** 10.3390/healthcare12181892

**Published:** 2024-09-21

**Authors:** Safiya Mahlaq, Ghizlane Rais, Redouane Abouqal, Jihane Belayachi

**Affiliations:** 1 Laboratory of Biostatistics, Clinical Research and Epidemiology (LBRCE), Faculty of Medicine and Pharmacy of Rabat, Mohammed V University, Rabat 10000, Morocco; r.abouqal@um5r.ac.ma (R.A.); jihanebelayachi@gmail.com (J.B.); 2 Medical Oncology Department, Biomed Laboratory, Faculty of Medicine and Pharmacy of Agadir, Ibn Zohr University, Agadir 80000, Morocco; g.rais@uiz.ac.ma

**Keywords:** cross-cultural adaptation, translation, psychometric validation, QLQ-SH22, cancer, sexual health, Morocco

## Abstract

Background: The Sexual Health Scale (QLQ-SH22) is the only cancer-specific measure of sexual health. It has never been translated into Arabic. In order to envisage effective healthcare strategies that improve sexual quality of life, the validation of the Moroccan version of this scale is a crucial step in exploring the influence of cancer and its treatment on patients in the Moroccan context. In this regard, this study aimed to validate a Moroccan Arabic version among patients with cancer. Method: A total of 280 Moroccan patients with cancer participated in this study from August 2022 to April 2023. The translation and cross-cultural adaptation of the QLQ-SH22 was performed following the EORTC guidelines. Psychometric validation was explored using the reliability of internal consistency, test–retest reliability, and confirmatory factor analyses (CFA). Results: The analysis revealed a greater internal consistency for both sexual satisfaction (α = 0.83) and sexual pain (α = 0.86). The intraclass correlation coefficient indicated an excellent level of test–retest reliability (from 0.925 to 0.993). The CFA demonstrated high-performing model fit indices (χ2/df = 1.17, SRMR = 0.05, RMSEA = 0.035, GFI = 0.94, CFI = 0.99, TLI = 0.99, IFI = 0.99, NFI = 0.94). The concurrent validity between the QLQ-C30 and QLQ-SH22 confirmed a strong correlation between the fatigue scales in both questionnaires (r = 0.69). This version showed good discrimination between known groups. Conclusions: The QLQ-SH22 Moroccan Arabic version has demonstrated a high level of reliability and validity, and therefore it is now ready for use.

## 1. Introduction

The effect of cancer and its treatment on sexuality has often been disregarded. The efficacy of the treatment has always been the priority, while the sexual health of the patient has been sacrificed. Only recently have the scientific, medical, and nursing communities become aware of the importance of the sexual dimension of cancer patients. Numerous empirical studies confirm the relevance of this issue. Consequently, the publication of international guidelines has aroused interest in the scientific community [1,2]. The observed results demonstrated an alarming impact on short- and long-term sexual health for both men and women, irrespective of the location of the cancer [3]. The results of the National Cancer Institute’s 2014 survey showed that 53.2% of participants experienced a decrease in their libido and even an absence of libido in 24% of cases [4]. A Moroccan study found that 97% of patients discontinued all sexual activity during treatment [5]. In addition, the negative impact of cancer on sexual health has been identified as an important predictor of global quality of life [6].

A couple’s relationship will certainly be influenced by these sexual disorders. Although the number of couples who separated was small, the majority suffered. Despite the availability of means to treat sexual problems in cancer patients, people are not prepared for intimate life experiences with this disease. The sexual dysfunction caused by cancer and its therapies need to be identified by healthcare staff and taught to patients [7].

Nevertheless, the quality of the couple relationship has been explored in patients with cancer. A better couple relationship was associated with better health indicators and decreased risk of mortality. In contrast, an unfavorable, negative conjugal partnership was strongly associated with a significant increase in the risk of mortality [8]. A fortiori, the authorities of societal, ethical, moral, and medical orders have appealed to cancer healthcare professionals to consider sexuality as a dimension of quality of life, by integrating it into the global, personalized healthcare management of oncology care [9]. 

The requirement to discuss sexuality in oncology includes several dimensions of morality, legality, and healthcare, following the logic that better sexual health contributes to a better global quality of life, thus promoting the healing process and predicting a better prognosis [10]. Hence, it is important to explore the repercussions of cancer on sexuality and to address these anomalies, preserve the couple and the family, and improve the psychological state of patients.

The cultural and societal aspect is an important determinant of healthcare. In Iran, discussions of sexuality are often considered inappropriate [11]. Sexual health is also a taboo subject in Moroccan culture. Although sexuality is a crucial dimension of quality of life, it is not sufficiently integrated in Moroccan clinical practice [5]. To enhance the articulation of concerns by cancer patients and facilitate discussions among healthcare professionals, it is crucial to have a Moroccan version of a validated instrument for measuring sexual health. This adaptation is essential for the effective demystification of this issue.

Sexual health is a broad concept that spans psychosexual to socio-behavioral dimensions, corresponding to the World Health Organization’s definition of sexual health, namely, a state of physical, emotional, mental, and social well-being linked to sexuality [12]. In this regard, during the Summit on Survival of the European Organisation for Research and Treatment of Cancer (EORTC), a team of researchers suggested, as a recommendation, backing the efforts of the Quality-of-Life Group (QLG) of the EORTC to create a well-designed instrument that will explore important sexual problems for cancer survivors, including physiological, psychological, and social aspects of sexuality [13]. To this end, a multidimensional sexual health scale for all types of cancer has recently been developed. The EORTC Quality of Life Questionnaire of Sexual Health (QLQ-SH22) has been approved in accordance with the EORTC QLG guidelines for questionnaire development and validation in four methodological phases, including the last phase of validation of psychometric properties, by an international study of validation of the scale structure [14].

Following an exhaustive review of the relevant literature, the questionnaire was developed and tested by healthcare professionals and patients from 13 different countries. The instrument was composed of 22 items and underwent a preliminary evaluation with a cross-cultural sample. Validation research was conducted with a sample of 444 individuals diagnosed with various types of cancer, at different stages of the disease and undergoing different forms of therapy. The hypothetical structure of the scale was confirmed through the analysis of two multi-item scales and eleven single items. The internal consistency of the scale was deemed acceptable, with Cronbach’s alpha coefficients of 0.80 and 0.90 [14,15].

The QLQ-SH22 is the most appropriate instrument for assessing clinical preoccupation. It is the only scale that measures sexual health that is specific to cancer patients. It has not been translated or validated into Arabic. This study aimed to adapt a Moroccan Arabic version of the QLQ-SH22 scale and explore its psychometric properties in Moroccan cancer patients.

## 2. Materials and Methods

### 2.1. Process of Translation and Cross-Cultural Adaptation

Cross-cultural adaptation was conducted in Moroccan Arabic. Considering Morocco’s linguistic diversity, Moroccan Arabic is the most widely spoken and comprehended language in the country. It serves as the native language for Arabic speakers and is also spoken by nearly half of the Amazigh population [16].

The translation and cross-cultural adaptation of the QLQ-SH22 was performed in five steps (initial translation, synthesis of translations, back-translation, expert committee review, and pilot test) according to the guidelines for translation and transcultural adaptation of the EORTC [17], and following methodological recommendations for the cross-cultural adaptation of questionnaires [18,19]. First, authorization for transcultural translation and adaptation was acquired from the Translation Unit (TU) of the EORTC, which prepared a rigorous step-by-step support file for this project. After the translation and back-translation stages, the Moroccan Arabic dialectal version was drafted and reviewed by an expert committee to assess the content validity of the questionnaire. This involved a qualitative evaluation of the items, which facilitated a conceptual deepening by discussing the relevance, clarity, comprehensiveness, and comprehensibility of the items and concepts. To this end, we consulted a panel of six experts (an oncologist, a gynecologist, a urologist, an epidemiologist, a sociologist, and a professor of higher education specializing in English) and four translators, all with more than 10 years of experience in their respective disciplines. In this step, the content validity was examined by the expert committees [20], which consisted of ensuring the stability of the content after the language change [21,22]. Next, face validity was checked during the pilot test stage; this was used to determine the understanding and pertinence of the instrument for the target population [23]. All reports, including the translations, back-translations, and the expert committee’s consensus on the prefinal version, were sent to the EORTC team for review and decision-making. The pre-final version was subsequently approved by the TU for pilot testing. Furthermore, the final version in Moroccan Arabic was written after a pilot pretest among ten patients diagnosed with cancer. The final version of the QLQ-SH22 in Moroccan Arabic was validated and addressed by the EORTC translation team and a certificate of completion of the process of translation and cross-cultural adaptation of the questionnaire was received.

### 2.2. Psychometric Validation

#### 2.2.1. Population and Study Design

This study employed a cross-sectional research design and was conducted in the Souss-Massa region of Morocco over a nine-month period (between August 2022 and April 2023). The target population was patients with all types of cancer followed at the regional oncology center of Agadir (public), which represents the principal destination for cancer patients in all areas of southern Morocco. Our inclusion criteria were as follows: being a Moroccan aged over 18 years, being married and living as a couple, being diagnosed with cancer, and having already started therapeutic healthcare. The exclusion criteria were as follows: being treated for psychiatric or mental problems, being in a critical state, being unable to practice any physical activity, and not understanding the Moroccan Arabic dialect. This study excluded participants with psychiatric or mental health illnesses to ensure accuracy and minimize confounding biases, as they are more likely to experience sexual dysfunction due to underlying illnesses or the use of psychotropic medication.

#### 2.2.2. Data Collection Instruments and Measurements

- Sociodemographic and clinical data

The questionnaire comprised two sections. The first addressed sociodemographic data, while the second part focused on clinical data. The socio-demographic data included the following: age, gender (male/female), living area (rural, urban), educational level (unschooled, primary, secondary, high school), occupational status (active, inactive, retired, loss of work through disease), and socioeconomic status (low, average, high). The socioeconomic status was defined in conformity with the classification system established by the Moroccan Haut Commissariat au Plan (HCP) [24,25].

Clinical characteristics included tumor site (breast/gynecologic/prostate/other genitourinary tumors/head and neck/colorectal/lungs/digestive/lymphoma and blood/others (liver, thyroid, etc.)), treatment (curative/palliative), disease status (new diagnosis/recurrence or progression/NED (no evidence of disease)), comorbidity (yes/no), and surgery (yes/no).

- Collection instrument

Performance status scale (ECOG): The “Eastern Cooperative Oncology Group” performance scale was used to evaluate the clinical status of patients. This unidimensional physical functioning scale measures the overall activity level of patients undergoing cancer treatment. The level of functionality was assessed by a healthcare professional using a score ranging from 0 (fully active) to 5 (dead). This provides five physical performance statuses for cancer survivors (completely active, limited activity, self-care possible, self-care limited, and self-care impossible) [26].

Quality-of-Life Questionnaire, core30 version 3.0 (QLQ-C30): The EORTC-QLQ C30 was used to assess the quality of life of cancer patients [27]. It consists of 30 items that establish the different dimensions of quality of life, including five functional dimensions (physical (items 1 to 5), role (items 6 and 7), cognitive (items 20 and 25), emotional (items 21 to 24), and social (items 26 and 27)), nine symptomatic dimensions (fatigue (items 10, 12, and 18), nausea and vomiting (items 14 and 15), pain (items 9 and 19), dyspnea (item 8), insomnia (item 11), appetite loss (item 13), constipation (item 16), diarrhea (item 17), and financial difficulties (item 28)), and a dimension measuring the global health status/QoL scale (items 29 and 30). All scales were linearly transformed into a score ranging from 0 to 100, where 0 corresponded to the worst quality of life and 100 corresponded to the highest score (“better”) of global health status/QOL or functional status. In terms of symptom scales, 0 corresponds to absence and 100 represents a high level (“worse”) of symptomatology/problems [28]. This questionnaire was valid and reliable for the assessment of quality of life in Morocco; Cronbach’s α was 0.87 for the total scale and ranged from 0.34 to 0.97 for the subscales [29].

The EORTC Quality of Life Questionnaire—Sexual Health (QLQ-SH22): The QLQ-SH22 incorporates two multi-item scales that assess sexual satisfaction (items 3, 4, 10, 12, 17, 18, 19, and 21) and sexual pain (items 8, 11, and 20). In addition, 11 single items assess sexual activity and explore treatment-related and partner-related matters: general questions about sexual health (sexual activity (item 1), decreased libido (item 2), incontinence (item 5), fatigue (item 6), treatment (item 7), communication with professionals (item 9), and partnership (item 13)) and 4 are gender-specific questions for men (confidence erections (item 14) and body image for men (item 15)) and women (body image for women (item 16) and vaginal dryness (item 22)). A high score indicated a high level of symptoms or problems. The scoring of the multi-item and single-item dimensions of the QLQ-SH22 is identical to that of the symptom scale dimensions of the QLQ-C30 [14,28].

#### 2.2.3. Sampling Procedure and Data Collection Process

Referring to the calculation method developed by David L. Streiner, according to the test–retest curve and for an intraclass correlation coefficient (ICC) of 0.75, the sample size for the test–retest analysis was 32 patients. According to the diagram illustrating the relation between the sample size and the number of items on the scale for a precision of ±0.10 and α = 0.70, the sample size for 22 items was almost 256 patients [30]. A sample size of 250 was considered sufficient to conduct a confirmatory factor analysis [31,32]. Based on the empirical method commonly used to calculate the sample size to validate psychometric qualities, a similar number of items multiplied by ten subjects is needed, that is, n = 220 per 22 items [33]. To compensate for the eventual losses, the estimated number will increase by 10%, resulting in a sample size of 262 patients. Finally, our sample size was 280 patients, 40 of whom participated in the test–retest process.

Patients were selected for participation during their follow-up visits and at treatment sessions, in accordance with the pre-established eligibility criteria. The above instruments were self-administered to educated patients. Those who were not educated were administered by the investigator (myself) of this research via an interview with the patients. The investigator was fully aware of the need to conduct interviews with unschooled patients in a neutral manner. This required reading the questions exactly as they were written and accurately recording the responses, with the aim of controlling the procedure as much as possible and ensuring that the data collected remained unbiased. All clinical data were collected from the patients’ medical records. The average time required to complete the questionnaire was approximately 10–15 min.

#### 2.2.4. Statistical Analysis

Descriptive statistics of sociodemographic and clinical data are presented as frequencies and percentages. Scale scores are presented as mean ± standard deviation and median and interquartile range (IQR).

In the context of clinical validity, we compared the different groups. Parametric or nonparametric tests were used for continuous variables, as appropriate, after the normality of the distribution was tested using the Shapiro–Wilk test. Statistical differences between two groups were determined by Student’s *t*-test or the Mann–Whitney U test, and differences between more groups were assessed by one-way ANOVA or the Kruskal–Wallis test.

In the order of concurrent validity, Pearson’s correlation or Spearman’s correlation was used to check the divergences and convergences between scores (continuous variables) of the two scales, QLQ-C30 and QLQ-SH22. Differences were considered statistically significant at a bilateral *p*-value < 0.05.

Reliability was assessed using internal consistency and test–retest reliability. Internal consistency was measured by Cronbach’s alpha coefficients and McDonald’s Omega coefficient for the multi-item dimensions. A Cronbach’s Alpha value > 0.7 may be interpreted as good internal reliability [34], and a McDonald’s Omega value > 0.8 may be regarded as indicative of satisfactory internal reliability [35]. Regarding test–retest reliability, a two-way random effects model with mean measurements in absolute agreement was used to measure the ICCs and their 95% confidence intervals. An ICC ranging from 0.75 to 0.9 indicates a good level of reliability, and a coefficient greater than 0.90 indicates an excellent level of reliability [34,35,36].

In terms of construct validity, the Kaiser–Meyer–Olkin (KMO) and Bartlett sphericity tests were performed as statistical evidence of data adequacy to carry out factor analysis. A KMO value ≥ 0.6 and a significance level of the Bartlett test of less than 0.05 are required for factor analysis to be appropriate [37,38].

Confirmatory factor analysis (CFA) is a method for verifying the degree of resemblance between the predefined theorical model and empirically collected data. If the results of the factorial analysis confirm the original model, the instrument is judged to be valid [23]; it was performed to verify the fit of the observed data to the predefined model of the original version. The assessment was performed using maximum likelihood estimates and the bootstrapping technique. The following criteria were adopted to assess the goodness of fit indices of the model—the absolute fit chi-square (χ2/df ≤ 2 or 3), Tucker–Lewis index (TLI), comparative fit index (CFI), incremental fit index (IFI), normalized fit index (NFI), and goodness-of-fit index (GFI)—with values higher than or equal to 0.90 indicating an acceptable fit and values higher than or equal to 0.95 indicating a good fit. The standardized root mean square (SRMS) with a value less than 0.08 indicated a good fit for the data. The root mean squared error of approximation (RMSEA) with a value less than 0.05 or 0.06 has been interpreted as an indicator of good fit [39,40,41]. Statistical analyses were performed using Jamovi software, version 2.2.5 [42,43,44,45].

## 3. Results

### 3.1. Sociodemographic and Clinical Characteristics of the Participants

A total of 280 patients was included in this study. Two thirds were women (67.1%), the mean age of the patients was 52.34 ± 10.54 years, and the ages ranged from 26 to 82 years. One hundred and eighty-six patients were unschooled, and approximately half (54.6%) were from urban areas. The majority (71.1%) had a low socioeconomic status, and 67.1% had no professional activity (Table 1).

The clinical characteristics of the study participants are presented in Table 2. One of the principal tumor sites was breast cancer: 53.9% of patients. A total of 180 patients (64.3%) were treated for curative purposes; 35.7% received palliative treatment. Two thirds (67.1%) of patients underwent surgical treatment. Most patients (68.6%) were newly diagnosed, 15.4% had NED, and 16% had recurrence or metastases. Physical performance was complete in thirty-one patients (11.1%) and decreased as follows: limited performance, possible self-care, and limited self-care in 35%, 37.5%, and 16.4% of patients, respectively.

### 3.2. Translation and Cross-Cultural Adaptation of the QLQ-SH22 Scale

In our study, we convened an expert committee to assess the content validity of the questionnaire. The panel of six consisted of experts in oncology, gynecology, urology, epidemiology, and sociology, and a professor of higher education specializing in English, all with over a decade of experience in their respective fields, including clinicians who regularly interact with patients. The investigator outlined the objectives of the meeting, the scope of the questionnaire, and the context of the translation, presenting both the original and the final translated versions. The experts collectively evaluated each item for relevance and clarity. Decisions regarding whether to retain, modify, or eliminate items were made by consensus, ensuring that the questionnaire content was aligned with the instrument’s objectives, with specific adjustments made where necessary. Notably, item 16 required refinement due to a phrase that needed clearer interpretation; “less feminine” was adjusted to the more accurate dialectal meaning of “to be as imperfect a woman.” Additionally, the term “sexual activity” was revised, as its translation into Moroccan dialect was ambiguous and required combining with another concept to clarify its meaning. This adjustment was agreed upon by consensus among the experts. All reports, including translations, back-translations, and the expert committee’s consensus on the pre-final version, were submitted to the EORTC team for review and decision-making. The pre-final version was then approved by the TU for pilot testing.

In the context of face validity, the pre-final Moroccan version of the QLQ-SH22 was administered to 10 cancer patients. The investigator utilized the response sheet and interview guide provided by the TU of the EORTC. The primary objective of the pilot test was to identify and address any potential translation issues by asking patients to highlight any difficulties they encountered in responding. Moreover, we aimed to identify any problematic terms or distressing expressions, and to determine whether patients might have preferred different wording, without altering the original item’s meaning but expressing it more clearly in Moroccan Arabic. The group of 10 patients had an average age of 48.8 years (ranging from 36 to 69 years). Four were men and six were women, with various cancer types (breast: 3, cervical: 1, colon: 2, prostate: 3, lung: 1). None of the 22 items posed significant challenges in terms of response or comprehension, and no patient suggested rephrasing any of the questions. After reviewing the outcomes of this phase, the TU of the EORTC finalized the questionnaire and provided a certificate confirming the completion of the process.

### 3.3. Acceptability

The findings of our empirical research demonstrated that all study participants (100%) agreed to complete the questionnaire, with no refusals. The schooled patients (33.6%) were able to complete the questionnaire independently. The investigator completed the questionnaire using the interviewing technique for unschooled patients (66.4%). The average time to complete the questionnaire was 10–15 min.

Some patients did not complete the five questions referring to sexual activity during the last four weeks (items 18, 19, 20, 21, and 22), as they were sexually inactive. The main reasons for sexual inactivity were fatigue (n = 40), physical deficiency (n = 30), neglect by the partner (n = 32), not interested in sex (n = 24), the partner not being interested in sex (n = 10), and having a physical problem (n = 2). Nevertheless, according to the QLQ-SH22 scoring manual [30], missing data due to sexual inactivity were treated separately and considered in the calculation of the scale scores. Therefore, for the dimension scores, there were no missing data except for the vaginal dryness score (n = 87).

### 3.4. Reliability

Internal consistency provided satisfactory values for the McDonald’s Omega coefficient: 0.84 for sexual satisfaction and 0.86 for sexual pain. Also, the Cronbach’s alpha coefficients were 0.83 for sexual satisfaction and 0.86 for sexual pain. The test–retest reliability measured by the ICC ranged from 0.925 to 0.993 for all dimensions of the scale (Table 3).

### 3.5. Validity

- Construct validity

The factorial structure of the QLQ-SH22 was examined by analyzing empirical data collected from the total sample using the QLQ-SH22, Moroccan Arabic version (n = 280). The adequacy of the sampling to perform this analysis was verified. The overall KMO value was 0.80, and the KMO value for the elements was greater than 0.60. Bartlett’s sphericity test (*p* < 0.001) indicated that inter-element correlations were sufficiently important to perform the factor analysis (see Table 4).

The factorial structure mentioned above was examined using CFA, and the model showed an excellent fit of the observed data with the theoretical model. All goodness-of-fit indices showed very convincing results (χ2/df = 1.17, RMSEA = 0.035, SRMR = 0.05, GFI = 0.94, FCI = 0.99, NFI = 0.94, IFI = 0.99, and TLI = 0.99) (Table 5).

**Figure 1:**
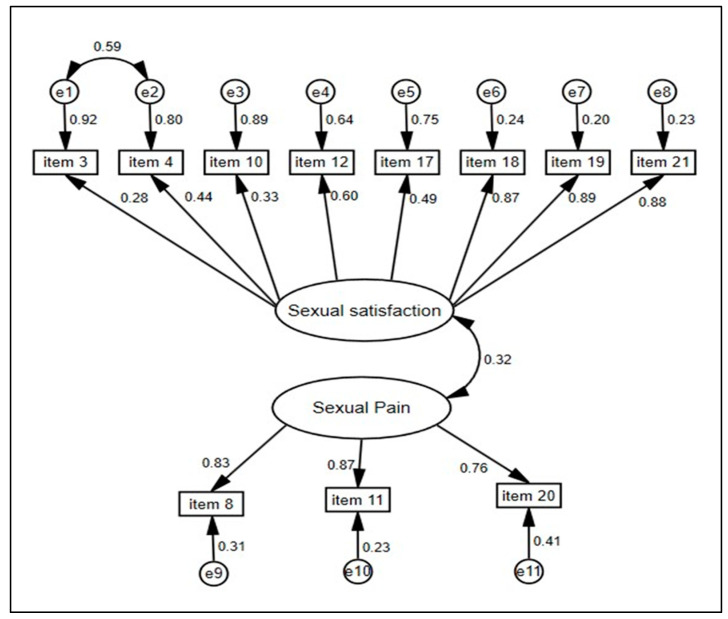
Path diagram of the AFC-derived model for muti-item scales, maximum likelihood estimation, and standardized estimates for associations and residuals (e). n = 280.

In addition, the CFA found that all factor loadings were statistically significant at the 5% level; the standardized parameter estimates are reported in Figure 1. The correlations between items and factors exceeded 0.60, except for items 3 and 10, which had r = 0.28 and r = 0.33, respectively. However, items 4 and 17 on the same dimension were extremely close to 0.50. This means that the latent variables may be measured by these elements. Moreover, CFA showed a low correlation (0.32) between the two multi-item dimensions. However, the QLQ-SH22 scale is multidimensional, which is the case for all quality-of-life scales. The sexual satisfaction dimension comprises eight items that assess patient satisfaction with diverse facets of sexuality. Item 3 evaluates satisfaction with sexual desire, while Item 10 assesses satisfaction with sexual communication. Although their correlations were relatively low, these items were conceptually representative of the dimension. The goodness of fit of the model demonstrates this.

- Concurrent validity

Regarding the concurrent validity of the QLQ-SH22 and QLQ-30 scales, the majority of the QLQ-SH22 scores were slightly correlated with the QLQ-C30 scale scores (r < 0.40). The most significant negative correlations were between the QLQ-C30 functioning scales and the QLQ-SH22 scores, namely, the QLQ-SH22 fatigue scale (physical functioning and fatigue, r = −0.38; role functioning and fatigue, r = −0.39) and the male body image score of QLQ-SH22 (physical functioning and male body image, r = −0.45; role functioning and male body image, r = −0.42). The most significant positive correlations were observed between the QLQ-C30 symptom score and the QLQ-SH22 scores, between the QLQ-C30 fatigue score and the QLQ-SH22 fatigue score (r = 0.69), and between the QLQ-C3O fatigue and the QLQ-SH22 treatment effect on sexual activity (r = 0.57) (see Supplement S1).

- Clinical validity

Comparisons between disease status groups showed that the problem of sexual satisfaction was significantly greater (*p* < 0.001) among patients with recurrence or progression of the disease (64.27 ± 16.35) and among newly diagnosed patients (61.01 ± 16.52) than among survivors with NED (45.85 ± 18.06). Also, symptom scores (decreased libido, fatigue, and effect of treatment on sexuality) were significantly (*p* < 0.001) lower in survivors in remission (50.39 ± 27.58, 48.06 ± 32.78, and 55.81 ± 30.62, respectively) than in the other two groups.

The problem of sexual activity was significantly (*p* < 0.001) greater among patients aged 65 years or older (50.00 ± 37.83) than among patients aged 35 years or younger (20.00 ± 16.90). Symptoms of decreased libido were more severe among patients aged 65 years or older (79.41 ± 23.23; *p* = 0.003). Moreover, the problem of communication with professionals was more common among patients aged 35 to 50 years than among those in other age groups (97.72 ± 9.51; *p* < 0.001). Male patients aged 36–50 years were less affected by the problem of confidence in their erection than were those aged 51–65 years (33.33 ± 22.47 vs. 58.70 ± 21.30, *p* < 0.001).

Regarding gender differences, the comparative analysis revealed that women reported higher levels of sexual pain than men (44.30 ± 26.75 vs. 33.33 ± 28.01, *p* = 0.001). In addition, the problem of sexual activity was less prevalent among men than among women (30.43 ± 31.12 vs. 43.97 ± 31.86, *p* < 0.001). Additionally, women suffered from the problem of sexual satisfaction more than men (61.42 ± 17.86 vs. 54.70 ± 16.44; *p* = 0.001).

Patients receiving palliative treatment had significantly (*p* = 0.012) higher decreased libido scores (75.33 ± 28.67) and significantly (*p* < 0.001) higher fatigue scores (87.67 ± 22.05) than patients undergoing curative treatment (67.22 ± 28.51 and 71.11 ± 29.35, respectively). Patients undergoing curative treatment had significantly less severe treatment effects (*p* = 0.008) than patients who underwent palliative treatment (72.41 ± 28.15 vs. 81.67 ± 23.39); body image dissatisfaction was greater in the palliative treatment group for women (67.97 ± 27.46; *p* = 0.019) and men (73.47 ± 31.17; *p* = 0.005).

Patients with a lower ECOG performance status and more severe treatment effects (80.13 ± 24.40) had significantly greater fatigue scores (85.21 ± 21.99; *p* < 0.001) than patients with a higher ECOG performance status (70.54 ± 28.76, *p* = 0.005). The problem of decreased libido was also significantly (*p* = 0.003) greater in patients with a low ECOG performance status (74.61 ± 27.94) than in patients with a higher ECOG performance status (64.86 ± 28.96). Patients with a low performance status had significantly greater sexual satisfaction problems (62.60 ± 16.72; *p* = 0.002) than patients with better ECOG performance status (55.24 ± 17.97) (refer to Supplement S2).

## 4. Discussion

Since the instrument is newly developed by the EORTC, its original validation has been very recent and it has never been subjected to cross-cultural validation. The QLQ-SH22 was subjected to cross-cultural adaptation and psychometric validation for the first time in our study, aiming to create an instrument with strong psychometric properties that can be applied in the Moroccan context to promote research on sexual health problems among Moroccan cancer patients.

In a cross-sectional study on a sample of Moroccan cancer patients, the QLQ-SH22 Moroccan Arabic version was explored in terms of reliability and validity on several levels. This scale comprises 13 dimensions with 2 multi-item dimensions of sexual satisfaction and sexual pain, and 11 dimensions with single items, including 7 items related to the patient’s sexual activity, partner, and sexual health (sexual activity, decreased libido, incontinence, fatigue, effect of treatment, communication with professionals, insecurity with partner) and 4 gender-specific items for men (erection confidence, body image (male)) and women (body image (female), vaginal dryness).

Overall, the psychometric quality analysis of the questionnaire produced satisfactory results compared to the methodological literature, and similar results to those of the original validation study of the QLQ-SH22 in 10 languages (Croatian, Danish, Dutch, French, German, Italian, Norwegian, Polish, Spanish, and Mandarin) [14].

The reliability of the QLQ-SH22 Moroccan version was evaluated using internal coherence and test–retest reliability. The internal consistency measured by Cronbach’s alpha for the two multi-item dimensions of sexual satisfaction and sexual pain were (α = 0.83, ω = 0.84) and (α = 0.86, ω = 0.86), respectively. These results indicate a good internal coherence of the scales [34,35,36]. In addition, the results of the validation study of the original version confirmed an internal consistency similar to our results (α = 0.90 and α = 0.80, respectively) for sexual satisfaction and sexual pain [14].

An ICC greater than 0.90 indicates excellent reproducibility [46]. Our test–retest reliability results, measured by the ICC, were between 0.925 and 0.993 for all dimensions of the scale, thus proving the excellent reproducibility and stability of the QLQ-SH22 Moroccan Arabic version. These results were similar to the test–retest reliability results of the original version as measured by Pearson’s correlation, which showed a strong correlation (from 0.70 to 0.93) for most dimensions [14].

The validity of the QLQ-SH22 Moroccan Arabic version was assessed using multiple methods, namely construct validity (CFA), concurrent validity, and clinical validity. Our empirical data had indices of sufficient quality (KMO = 0.80, Bartlett test < 0.001), well above the acceptable limit of 0.60 [38], ensuring the adequacy of the sample for factor analysis.

The CFA demonstrated good model fit indices, concluding that the Arabic dialect version conformed to the original version of the QLQ-SH22. The model fit indices in the original validation study (GFI = 0.98; SRMR = 0.07) were similar to those in the present study (GFI = 0.94; SRMR = 0.05). Other indices of model fit quality were explored in this study: χ2/df = 1.17, RMSEA = 0.035, CFI = 0.99, TLI = 0.99, IFI = 0.99, and NFI = 0.94. According to the literature, these results confirm the excellent fit of the dialectal Arabic version with the original version of the QLQ-SH22 [40,47,48].

Regarding the concurrent validity of the QLQ-C30 and QLQ-SH22, the results of the correlation analysis demonstrated reasonable convergence and divergence between the scores of the dimensions of the two measurement scales, which confirmed that the concurrent validity was congruent according to the measurement interest of the dimensions. The QLQ-SH22 was developed as a stand-alone measurement, and most of these dimensions were different from those evaluated by the QLQ-C30. Only the fatigue dimension appeared in the two questionnaires; therefore, the strongest correlation (r = 0.69; *p* < 0.001) was between the fatigue dimensions of QLQ-SH22 and QLQ-C30. These observations were corroborated by the results of the original validation [14]. The QLQ-SH22 is a symptom scale [14,28]. Consequently, several significant positive correlations have been found between these scores and QLQ-C30 symptom scores, indicating a convergent effect. Additionally, divergences were confirmed by significant negative correlations between QLQ-C30 functional scores and QLQ-SH22 scores.

According to the results of our study, the QLQ-SH22 Moroccan Arabic version showed good discrimination between known groups. The analysis of comparisons confirmed the desired differences; for example, the problem of sexual satisfaction, decreased libido, fatigue, and the effect of the treatment on sexuality differed according to disease status. For men, the intention of treatment and age discriminated against sexual activity, decreased libido and erectile confidence. Several comparisons of QLQ-SH22 scores stratified by gender and by physical performance status (ECOG) were logically significant. Discrimination between known groups revealed that the QLQ-SH22 Moroccan Arabic version is clinically valid. This finding was similar to the results of the original validation, which showed good discrimination between patient subgroups [14].

The Moroccan Arabic version was perfectly reliable and valid in the Moroccan context and is ready for potential utilization. The QLQ-SH22 Moroccan Arabic version will be used by researchers and health professionals to address sexual health disorders related to cancer and its treatment in the Moroccan context. In this way, it will help Moroccan cancer patients better communicate their sexual health problems and help Moroccan healthcare professionals understand the concerns of cancer patients regarding this taboo subject.

## 5. Strengths and Limitations of the Study

One of the strengths of our study is that it was carried out in a regional center of oncology. According to the territorial division of the kingdom, this center covers the entire southern region, which includes four of the twelve regions of Morocco. Another strength of our study is that it was the first study in the world to carry out the translation and cross-cultural adaptation of the QLQ-SH22. This is also the first study to evaluate its psychometric properties in the Moroccan context. Nevertheless, a limitation is that our study included data from only one regional center. However, the validation of the QLQ-SH22 Moroccan Arabic version in different regions of Morocco is recommended. Furthermore, the phenomenon of sexual health, as measured by the QLQ-SH22, is culturally linked to the problem of socially desirable responses, which impose inherent limits. In discussing this subject with patients, it is essential that the conditions be created for comfort and trust. Additionally, reliance on self-reporting introduces the problem of subjectivity inherent in any self-report study. It is therefore imperative that studies consider these factors in order to reduce their potential impact on research findings.

## 6. Conclusions

In conclusion, this study represents the first validation of the QLQ-SH22 scale in the Moroccan Arabic version. Its translation and cross-cultural adaptation were conducted under the guidance of the EORTC team. After validation of the Arabic dialect version by this team, psychometric properties were evaluated in a sample of 280 Moroccan cancer patients. It has been claimed that the QLQ-SH22 Moroccan Arabic version has a structure identical to that of the original version, with 22 items, and 13 dimensions, including 2 multi-item dimensions and 11 single-item dimensions. Additionally, the QLQ-SH22 Moroccan Arabic version demonstrated a high level of reliability in terms of internal consistency and stability. Also, the results proved clinical and concurrent validity.

The QLQ-SH22 Moroccan Arabic version has demonstrated interesting characteristics and excellent psychometric qualities, paving the way for its use in clinical research among cancer patients and in clinical settings to facilitate the assessment of sexual health by healthcare professionals. The validated QLQ-SH22 Moroccan Arabic version may be used to evaluate sexual health, demystify the repercussions of cancer on sexual health in cancer patients in Morocco, and recommend improvements and solutions affording holistic healthcare.

## Figures and Tables

**Table 1:** Sociodemographic characteristics.

Characteristic	No. of Patients (%)
Age (years), mean (SD)	52.34 ± 10.54 (range: 26–82)
<50 years old	132 (47.1)
≥50 years old	148 (52.9)
Gender	
Female	188 (67.1)
Male	92 (32.9)
Socioeconomic status	
Low	199 (71.1)
Middle	74 (26.4)
High	7 (2.5)
Educational status	
Unschooled	186 (66.4)
Primary	48 (17.1)
Secondary	43 (15.4)
Higher	3 (1.1)
Occupation status	
Active	21 (7.5)
Inactive	188 (67.1)
Retired	10 (3.6)
Loss of work	61 (21.8)
Prevenance	
Rural	127 (45.4)
Urban	153 (54.6)

**Table 2:** Clinical characteristics.

Characteristic	No. of Patients (%)
Tumor site	
Breast	151 (53.9)
Gynecologic	22 (7.9)
Prostate	6 (2.1)
Other genitourinary	6 (2.1)
Head and neck	8 (2.9)
Colorectal	17 (6.1)
Lung	22 (7.9)
Digestive	13 (4.6)
Lymphoma and blood	28 (10.0)
Other (liver, thyroid, etc.)	7 (2.5)
Treatment	
Curative	180 (64.3)
Palliative	100 (35.7)
Status of disease	
NED	43 (15.4)
Newly diagnosed	192 (68.6)
Recurrence/progression	45 (16)
Comorbidity	
No	199 (71.1)
Yes	81 (28.9)
ECOG	
Fully active	31 (11.1)
Restricted	98 (35.0)
Self-care possible	105 (37.5)
Limited self-care	46 (16.4)
Surgery	
No	92 (32.9)
Yes	188 (67.1)

Abbreviation: NED (No Evidence of Disease); ECOG (Eastern Cooperative Oncology Group).

**Table 3:** Results of the reliability analysis of the EORTC QLQ-SH22.

Scale	Items	Mean	SD	Median	Interquartile	Cronbach’s Alpha α	McDonald’s Omega ω	Test–Retest ICC (n = 40)	Valid n
Multi-item scales									
Sexual satisfaction	8	2.78	0.53	2.80	(2.5; 3.13)	0.828	0.844	0.984(0.970; 0.991)	280
Sexual pain	3	2.22	0.83	2.00	(1.67; 2.67)	0.862	0.864	0.993(0.986; 0.996)	280
Single-item scales									
Importance of sexual activity	1	2.18	0.97	2.00	(1.00; 3.00)			0.942(0.890; 0.969)	280
Decreased libido	1	3.10	0.86	3.00	(2.00; 4.00)			0.988(0.978; 0.994)	280
Worry incontinence	1	1.35	0.59	1.00	(1.00; 2.00)			0.988(0.977; 0.993)	280
Fatigue	1	3.32	0.84	4.00	(3.00; 4.00)			0.936(0.880; 0.966)	280
Treatment effect on sexual activity	1	3.27	0.81	3.00	(3.00; 4.00)			0.960(0.924; 0.979)	280
Communication with professionals	1	3.82	0.47	4.00	(4.00; 4.00)			0.966(0.936; 0.982)	280
Insecurity with the partner	1	2.65	0.84	3.00	(2.00; 3.00)			0.969(0.942; 0.984)	280
Erectile dysfunction	1	2.48	0.72	2.00	(2.00; 3.00)			0.972(0.939; 0.987)	92
Body image (male)	1	2.96	0.96	3.00	(2.00; 4.00)			0.981(0.959; 0.991)	92
Body image (female)	1	2.78	0.92	3.00	(2.00; 3.00)			0.968(0.900; 0.990)	188
Vaginal dryness	1	2.47	0.86	3.00	(2.00; 3.00)			0.925(0.717; 0.980)	101

Abbreviations: ICC (Intraclass Correlation Coefficient); SD (standard deviation).

**Table 4:** Results of KMO (Kaiser–Meyer–Olkin) of the EORTC QLQ-SH22.

Component	KMO (Kaiser–Meyer–Olkin)
Item 3: Have you been satisfied with your level of sexual desire?	0.60
Item 4: Has sexual activity been enjoyable for you?	0.67
Item 10: Have you been satisfied with your ability to reach an orgasm?	0.83
Item 12: Have you been satisfied with the communication about sexual issues between yourself and your partner?	0.91
Item 17: Have you been satisfied with your level of intimacy?	0.89
Item 18: Have you been sexually active?	0.86
Item 19: To what extend did you feel sexual enjoyment?	0.83
Item 21: Have you been satisfied with your sex life?	0.86
Item 8: Have you felt pain during/after sexual activity?	0.76
Item 11: Have you been worried that sex would be painful?	0.73
Item 20: Have you been worried that your partner may cause you pain during sexual contact?	0.80
KMO overall	0.80

**Table 5:** Results of the confirmatory factor analysis of the EORTC QLQ-SH22.

Indices of Goodness of Fit Statistics	Model
Chi-square test/degrees of freedom (χ2/df)	1.17
Tucker–Lewis Index (TLI)	0.99
Fit Comparative Index (FCI)	0.99
Goodness-of-Fit Index (GFI)	0.94
Incremental Fit Index (IFI)	0.99
Normalized Fit Index (NFI)	0.94
Standardized Root Mean Residue (SRMR)	0.05
Root Mean Squared Error of Approximation (RMSEA)	0.035

## Data Availability

The datasets used and/or analyzed during the current study are available from the corresponding author upon reasonable request.

## Supplementary Materials

The following supporting information can be downloaded at: https://www.mdpi.com/article/10.3390/healthcare12181892/s1. Supplementary Material S1: Correlations between the EORTC QLQ-SH22 and the EORTC QLQ-C30. Supplementary Material S2: Comparison analysis of QLQ-SH22 scores between known groups.

## References

[1] Hordern A. (2008). Intimacy and Sexuality After Cancer: A Critical Review of the Literature. Cancer Nurs..

[2] Nuytten M., Faugeras L., D’Hondt L. (2018). Cancer et Sexualité. Louvain Med..

[3] Schweizer A., Toffel K., Braizaz M. (2021). L’abord de La Sexualité Par Les Professionnel·le·s de Santé En Oncologie: Une Revue de La Littérature. Psychol. Fr..

[4] INCa (2014). «La vie deux ans après un Diagnostic de cancer—De L’annonce à l’après Cancer », Collection Études et Enquêtes.

[5] Dahbi Z., Sbai A., Mezouar L. (2018). Sexuality of Moroccan Survivors of Cervical Cancer: A Prospective Data. Asian Pac. J. Cancer Prev. APJCP.

[6] Ismaili R., Nejmeddine A., Mimouni H., El Haouachim I., Hilali A., Rahou B., Bekkali R., Loukili L. (2022). The Impact of Sexual Life Determinants on the Quality of Life of Moroccan Breast and Lung Cancer Survivors Two Years after Diagnosis. Univers. J. Public Health.

[7] Bondil P., Habold D. (2015). Développement de l’oncosexualité et de l’oncofertilité en France: Pourquoi maintenant ? Aspects culturels et psychosociologiques. Psycho-Oncologie.

[8] Roy V. (2021). Le rôle Atténuateur du Soutien Social Dans l’effet du Stress sur le Fonctionnement Immunitaire et les Infections chez des Femmes Traitées en Chimiothérapie Pour un Cancer du sein. https://corpus.ulaval.ca/server/api/core/bitstreams/ce5ade0c-19fc-42c2-8bce-34b4eb69afca/content.

[9] Bondil P., Habold D., Carnicelli D. (2016). Cancer et Sexualité: Le Couple, Un Déterminant Trop Souvent Négligé. Sexologies.

[10] Moreau É., Moulin P., Giami A. (2016). L’évolution des liens entre cancer et sexualité: Revue critique de la littérature. Psycho-Oncologie.

[11] Mardani A., Farahani M.A., Khachian A., Maleki M., Vaismoradi M. (2024). Qualitative Exploration of Sexual Dysfunction and Associated Coping Strategies among Iranian Prostate Cancer Survivors. Support. Care Cancer.

[12] World Health Organization (2006). Report of Technical Consultation on Sexual Health 28–31 January 2002.

[13] Schover L.R., Van Der Kaaij M., Van Dorst E., Creutzberg C., Huyghe E., Kiserud C.E. (2014). Sexual Dysfunction and Infertility as Late Effects of Cancer Treatment. Eur. J. Cancer Suppl..

[14] Greimel E., Nagele E., Lanceley A., Oberguggenberger A.S., Nordin A., Kuljanic K., Arraras J.I., Wei-Chu C., Jensen P.T., Tomaszewski K.A. (2021). Psychometric Validation of the European Organisation for Research and Treatment of Cancer–Quality of Life Questionnaire Sexual Health (EORTC QLQ-SH22). Eur. J. Cancer.

[15] Oberguggenberger A.S., Nagele E., Inwald E.C., Tomaszewski K., Lanceley A., Nordin A., Creutzberg C.L., Kuljanic K., Kardamakis D., Schmalz C. (2018). Phase 1-3 of the Cross-Cultural Development of an EORTC Questionnaire for the Assessment of Sexual Health in Cancer Patients: The EORTC SHQ-22. Cancer Med..

[16] Chatar-Moumni N. (2015). Vers une standardisation de l’arabe marocain?. Echo Études Romanes.

[17] Kuliś D., Bottomley A., Velikova G., Greimel E., Koller M. (2017). EORTC Quality of Life Group Translation Procedure.

[18] Beaton D.E., Bombardier C., Guillemin F., Ferraz M.B. (2000). Guidelines for the Process of Cross-Cultural Adaptation of Self-Report Measures. Spine.

[19] Guillemin F., Bombardier C., Beaton D. (1993). Cross-Cultural Adaptation of Health-Related Quality of Life Measures: Literature Review and Proposed Guidelines. J. Clin. Epidemiol..

[20] Mokkink L.B., de Vet H.C.W., Prinsen C.A.C., Patrick D.L., Alonso J., Bouter L.M., Terwee C.B. (2018). COSMIN Risk of Bias Checklist for Systematic Reviews of Patient-Reported Outcome Measures. Qual. Life Res..

[21] Coste J., Fermanian J., Venot A. (1995). Methodological and Statistical Problems in the Construction of Composite Measurement Scales: A Survey of Six Medical and Epidemiological Journals. Stat. Med..

[22] Fermanian J. (1996). Evaluating correctly the validity of a rating scale: The numerous pitfalls to avoid. Rev. Epidemiol. Sante Publique.

[23] Fayers P.M., Machin D. (2016). Quality of Life: The Assessment, Analysis, and Reporting of Patient-Reported Outcomes.

[24] Maaroufi Y. Etude sur les Classes Moyennes au Maroc. https://www.hcp.ma/Etude-sur-les-classes-moyennes-au-Maroc_a780.html.

[25] Maaroufi Y. Pauvreté et Prospérité Partagée au Maroc du Troisième Millénaire, 2001–2014. https://www.hcp.ma/Pauvrete-et-prosperite-partagee-au-Maroc-du-troisieme-millenaire-2001-2014_a2055.html.

[26] Oken M.M., Creech R.H., Tormey D.C., Horton J., Davis T.E., McFadden E.T., Carbone P.P. (1982). Toxicity and Response Criteria of the Eastern Cooperative Oncology Group. Am. J. Clin. Oncol..

[27] Aaronson N.K., Ahmedzai S., Bergman B., Bullinger M., Cull A., Duez N.J., Filiberti A., Flechtner H., Fleishman S.B., de Haes J.C.J.M. (1993). The European Organization for Research and Treatment of Cancer QLQ-C30: A Quality-of-Life Instrument for Use in International Clinical Trials in Oncology. JNCI J. Natl. Cancer Inst..

[28] Fayers P.M., Aaronson N., Bjordal K., Groenvold M., Curran D., Bottomley A. (2001). EORTC QLQ-C30 Scoring Manual: This Manual Is Intended to Assist Users with Scoring Procedures for the QLQ-C30 Version 3 and Earlier, and the QLQ Supplementary Modules.

[29] Nejjari C., El Fakir S., Bendahhou K., El Rhazi K., Abda N., Zidouh A., Benider A., Errihani H., Bekkali R. (2014). Translation and Validation of European Organization for Research and Treatment of Cancer Quality of Life Questionnaire -C30 into Moroccan Version for Cancer Patients in Morocco. BMC Res. Notes.

[30] Streiner D.L., Norman G.R., Cairney J. (2015). Health Measurement Scales: A Practical Guide to Their Development and Use.

[31] Cattell R. (2012). The Scientific Use of Factor Analysis in Behavioral and Life Sciences.

[32] Guadagnoli E., Velicer W.F. (1988). Relation of Sample Size to the Stability of Component Patterns. Psychol. Bull..

[33] Jackson D.L. (2003). Revisiting Sample Size and Number of Parameter Estimates: Some Support for the N:Q Hypothesis. Struct. Equ. Model. Multidiscip. J..

[34] Cronbach L.J. (1951). Coefficient Alpha and the Internal Structure of Tests. Psychometrika.

[35] Feißt M., Hennigs A., Heil J., Moosbrugger H., Kelava A., Stolpner I., Kieser M., Rauch G. (2019). Refining Scores Based on Patient Reported Outcomes—Statistical and Medical Perspectives. BMC Med. Res. Methodol..

[36] McDonald R.P. (2013). Test Theory: A Unified Treatment.

[37] Guttman L. (1954). Some Necessary Conditions for Common-Factor Analysis. Psychometrika.

[38] Tabachnick B.G., Fidell L.S., Ullman J.B. (2013). Using Multivariate Statistics.

[39] Hair J.F., Babin B.J., Krey N. (2017). Covariance-Based Structural Equation Modeling in the Journal of Advertising: Review and Recommendations. J. Advert..

[40] Hu L., Bentler P.M. (1999). Cutoff Criteria for Fit Indexes in Covariance Structure Analysis: Conventional Criteria versus New Alternatives. Struct. Equ. Model. Multidiscip. J..

[41] Schreiber J.B. (2008). Core Reporting Practices in Structural Equation Modeling. Res. Soc. Adm. Pharm..

[42] (2024). The Jamovi Project. https://www.Jamovi.Org.

[43] R Core Team (2023). R: A Language and Environment for Statistical Computing. https://Cran.r-Project.Org.

[44] Gallucci M., Jentschke S. (2021). SEMLj: Jamovi SEM Analysis. https://Semlj.Github.Io/.

[45] Epskamp S., Stuber S., Nak J., Veenman M., Jorgensen T.D. semPlot: Path Diagrams and Visual Analysis of Various SEM Packages’ Output 2022. https://CRAN.R-project.org/package=semPlot.

[46] Koo T.K., Li M.Y. (2016). A Guideline of Selecting and Reporting Intraclass Correlation Coefficients for Reliability Research. J. Chiropr. Med..

[47] Costello A., Osborne J. (2019). Best Practices in Exploratory Factor Analysis: Four Recommendations for Getting the Most from Your Analysis. Pract. Assess. Res. Eval..

[48] Berger J.-L. Analyse Factorielle Exploratoire et Analyse En Composantes Principales: Guide Pratique 2021. https://hal.science/hal-03436771/document.

