# Supplementary material for: Cross-Cultural Adaptation and Pilot Psychometric Validation of the European Organisation for Research and Treatment of Cancer—Quality of Life Questionnaire—Sexual Health (EORTC QLQ-SH22) Scale, Moroccan Arabic Version
Source: Healthcare (Basel). 2024 Sep 21;12(18):1892. doi: 10.3390/healthcare12181892 (PMC11431793; doi:10.3390/healthcare12181892)
Supplement: Supplementary file 1 [file healthcare-12-01892-s001.zip › Supplement S1.pdf]

Supplement S1: Correlations between the EORTC QLQ-SH22 and the EORTC QLQ-C30

Table S1: Correlations between the EORTC QLQ-SH22 and the EORTC QLQ-C30.

Scales	QLQ-SH22[1]												
	SXSAT	SXP	SXA	DLI	WI	FA	TX	CHCP	ISP	CE	BIM	VD	BIF
**QLQ-C30**													
Physical functioning	-0.24**	-0.08	-0.09	-0.17**	-0.17**	**-0.38****	-0.26**	0.07	-0.19**	-0.25*	**-0.45****	-0.29**	-0.16*
Role functioning	-0.25**	-0.21**	-0.09	-0.21**	-0.07	**-0.39****	**-0.38****	0.02	-0.29**	**-0.34****	**-0.42****	-0.14	-0.19**
Emotional functioning	-0.25**	-0.01	-0,03	-0.18**	0.02	-0.10	-0.12*	-0.04	-0.12*	-0.15	0.03	-0.22*	-0.08
Cognitive functioning	-0.19**	0.03	-0.02	-0.08	-0.14*	-0.09	-0.06	0.05	0.02	-0.31**	-0.25*	-0.33**	-0.05
Social functioning	-0.15*	0.08	-0.07	-0.04	0.03	-0.02	0.002	-0.03	-0.03	-0.11	-0.05	-0.20*	-0.10
Global health status	0.14**	0.03	-0.01	0.02	0.08	0.19**	0.10	-0.05	0.02	-0.01	0.005	0.26**	0.14
Fatigue	0.29**	0.26**	0.26**	0.35**	0.17*	**0.69****	**0.56****	-0.01	0.23**	0.16	**0.37****	0.23*	0.07
Nausea and vomiting	0.27**	0.14*	0.15**	0.12*	-0.01	0.18**	0.23**	0.01	0.15**	0.02	0.24*	**0.33****	0.05
Pain	0.28**	**0.32****	0.07	0.24**	0.14*	0.23**	0.23**	-0.05	0.20**	0.15	0.32**	0.31**	0.13
Dyspnea	0.19**	0.14*	0.13*	0.15**	0.09	0.21**	0.17**	-0.06	0.16**	0.15	0.19	**0.39****	0.13
Insomnia	0.29**	0.17**	0.12*	0.11	0.13*	0.10	0.08	0.01	0.17**	0.27*	0.26*	**0.41****	0.03
Appetite loss	**0.32****	0.16**	0.09	0.17**	0.02	0.27**	0.22**	0.09	0.15*	0.26*	**0.34****	0.27*	0.03
Constipation	0.09	-0.004	-0.10	0.13*	0.07	0.11	0.03	-0.11	0.03	0.29**	0.23*	0.19	0.13
Diarrhea	-0.03	0.04	0.07	-0.05	0.03	-0.07	-0.08	-0.03	0.02	0.13	-0.003	-0.02	-0.01
Financial difficulties	-0.003	-0.08	-0.02	-0.02	0.00	0.07	0.03	-0.09	-0.05	0.25*	-0.03	-0.09	-0.03

*p < .05, **p < .01. [1] Spearman's correlation coefficient

SXSA, sexual satisfaction; SXP, sexual pain; ISXA, importance of sexual activity; DLI, decreased libido; WI, worry incontinence; FA, fatigue; TX, treatment effect on sexual activity; CHCP, communication with professionals; ISP, insecurity with the partner; CE, confidence erection; BIM, body image (male); VD, vaginal dryness; BIF, body image (female); EORTC QLQ-SH22, The European Organisation for Research and Treatment of Cancer Quality of Life Questionnaire of Sexual Health.
