# Supplementary material for: Cross-Cultural Adaptation and Pilot Psychometric Validation of the European Organisation for Research and Treatment of Cancer—Quality of Life Questionnaire—Sexual Health (EORTC QLQ-SH22) Scale, Moroccan Arabic Version
Source: Healthcare (Basel). 2024 Sep 21;12(18):1892. doi: 10.3390/healthcare12181892 (PMC11431793; doi:10.3390/healthcare12181892)
Supplement: Supplementary file 1 [file healthcare-12-01892-s001.zip › Supplement S2.pdf]

Supplement S2: Comparisons analysis of QLQ-SH22 scores between known groups.

Table S2. Differences in the QLQ-SH22 scales by sex

Dimensions	Females			Males			P*
	M±SD	Median (interquartile range)	N	M±SD	Median (interquartile range)	N	
Sexual satisfaction	61.42±17.86	60.00 (53.33; 73.33)	188	54.70±16.44	53.33(46.67; 66.67)	92	**0.001**
Sexual pain	44.30±26.75	33.33 (33.33; 58.34)	188	33.33±28.01	33.33(0.00; 50.00)	92	**0.003**
Importance of sexual activity	43.97±31.86	33.33(33.33; 66.67)	188	30.43±31.12	33.33(0.00; 66.67)	92	**< .001**
Decreased libido	71.63±28.20	66.67(58.33; 100.00)	188	67.03±29.85	66.67(33.33; 100.00)	92	0.251
Worry incontinence	11.17±18.54	0.00(0.00; 33.33)	188	12.68±22.01	0.00(0.00; 33.33)	92	0.881
Fatigue	77.66±28.17	100.00(66.67; 100.00)	188	75.72±28.00	66.67(66.67; 100.00)	92	0.474
Treatment effect on sexual activity	78.72±25.73	100.00(66.67; 100.00)	188	69.57±28.24	66.67(66.67; 100.00)	92	**0.007**
Communication with professionals	96.45±11.91	100.00(100.00; 100.00)	188	89.13±21.02	100.00(91.67; 100.00)	92	**< .001**
Insecurity with the partner	57.27±27.59	66.67(33.33; 66.67)	188	50.00±28.60	66.67(33.33; 66.67)	92	0.070
Confidence erection	n.a	n.a	0	49.28±23.95	33.33(33.33; 66.67)	92	-
Body image (male)	n.a	n.a	0	65.22±31.99	66.67(33.33; 100.00)	92	-
Body image (female)	59.220±30.70	66.67(33.33; 66.67)	188	n.a	n.a	0	-
Vaginal dryness	49.17±28.52	66.67(33.33; 66.67)	101	n.a	n.a	0	-

n.a : not applicable; * Mann–Whitney U test

Table S3. Differences in the QLQ-SH22 scales by age

Dimensions	≤35 years			36-50 years			51-65 years			>65 years			P*
	M±SD	Median (interquartile range)	N	M±SD	Median (interquartile range)	N	M±SD	Median (interquartile range)	N	M±SD	Median (interquartile range)	N	
Sexual satisfaction	44.89±21.18	50.00(33.33; 57.08)	15	58.50±17.88	58.33(50.00;70.83)	117	60.92±17.03	60.00(50.00;73.33)	114	62.25±14.65	60.00(53.33; 73.33)	34	0.061
Sexual pain	40.00±29.41	33.33(19.44; 61.11)	15	41.45±27.11	33.33(22.22;55.56)	117	40.06±27.84	33.33(22.22;55.56)	114	40.52±28.90	33.33(33.33; 50.00)	34	0.965
Importance of sexual activity	20.00±16.90	33.33(0.00; 33.33)	15	32.48±30.15	33.33(0.00; 33.33)	117	46.20±31.52	33.33(33.33;66.67)	114	50.00±37.83	66.67(8.33; 66.67)	34	**< .001**
Decreased libido	55.56±32.53	66.67(50.00;66.67)	15	64.39±30.24	66.67(33.33;100.00)	117	75.15±26.54	66.67(66.67;100.00)	114	79.41±23.23	83.33(66.67;100.0)	34	**0.003**
Worry incontinence	4.44±11.728	0.00(0.00;0.00)	15	8.546±16.47	0.00(0.00;0.00)	117	14.03±21.22	0.00(0.00 ;33.33)	114	17.65±24.94	0.00 (0.00;33.33)	34	**0.033**
Fatigue	62.22±35.34	66.67(33.33;100.00)	15	75.21±28.42	100.00(66.67;100.00)	117	78.95±26.70	100.00(66.67;100.00)	114	83.33±26.27	100.00(66.67;100.00)	34	0.110
Treatment effect on sexual activity	66.67±35.63	66.67(50.00;100.00)	15	75.78±26.85	66.67(66.67;100.00)	117	76.61±25.06	66.67(66.67;100.00)	114	76.47±29.05	83.33(66.67;100.00)	34	0.813
Communication with professionals	82.22±27.79	100.00(66.67;100.00)	15	97.72±9.51	100.00 (100.00;100.00)	117	91.23±18.84	100.00(100.00;100.00)	114	96.08±10.90	100.00(100.00;100.0)	34	**< .001**
Insecurity with the partner	55.56±32.53	66.67(33.33;66.67)	15	56.13±27.91	66.67(33.33 ; 66.67)	117	54.68±26.66	66.67(33.33 ; 66.67)	114	50.98±32.03	50.00 (33.33; 66.67)	34	0.816
Confidence erection	38.89±13.61	33.33(33.33;33.33)	6	33.33±22.47	33.33(33.33;33.33)	23	58.70±21.30	66.67(33.33;66.67)	46	49.02±23.92	33.33(33.33;66.67)	17	**< .001**
Body image (male)	50.00±27.89	66.67(41.66;66.67)	6	53.62±32.94	66.67(33.33;66.67)	23	68.84±31.74	66.67(33.33;100.00)	46	76.47±28.30	66.67(66.67;100.00)	17	0.064
Body image (female)	51.85±41.20	33.33(33.33;100.00)	9	63.48±27.68	66.67(33.33;66.67)	94	55.88±30.71	66.67(33.33;66.67)	68	52.94±39.19	66.67(33.33;100.00)	17	0.373
Vaginal dryness	27.78±25.09	33.33(8.33;33.33)	6	50.75±26.82	66.67(33.33;66.67)	67	48.72±31.60	66.67(33.33;66.67)	26	66.66±47.14	66.67 (50.00;83.33)	2	0.244

*Kruskal–Wallis test

Table S4. Differences in the QLQ-SH22 scales by state of disease

Dimensions	Newly diagnosed			No evidence of disease			Recurrence/progression			P*
	M±SD	Median (interquartile range)	N	M±SD	Median (interquartile range)	N	M±SD	Median (interquartile range)	N	
Sexual satisfaction	61.01±16.52	60.00(50.00; 73.33)	192	45.85±18.06	46.67(33.33; 62.50)	43	64.27±16.35	62.50(54.17; 70.83)	45	**< .001**
Sexual pain	40.51±26.62	33.33(22.22; 55.56)	192	35.40±27.32	33.330(13.89;50.00)	43	46.54±31.32	44.44(33.33; 66.67)	45	0.200
Importance of sexual activity	40.62±32.07	33.33(0.00; 66.67)	192	33.33±33.33	33.33(0.00; 50.00)	43	40.74±31.69	33.33(33.33; 66.67)	45	0.312
Decreased libido	72.22±27.78	66.67(66.67; 100.00)	192	50.39±27.58	33.33(33.33; 66.67)	43	80.00±26.01	100.00(66.67; 100.00)	45	**< .001**
Worry incontinence	12.85±20.67	0.00(0.00; 33.33)	192	7.75±16.00	0.00(0.00; 0.00)	43	10.37±18.56	0.00(0.00; 33.33)	45	0.289
Fatigue	81.08±24.25	100.00(66.67; 100.00)	192	48.06±32.78	33.33(33.33; 66.67)	43	87.41±20.46	100.00(66.67; 100.00)	45	**< .001**
Treatment effect on sexual activity	77.95±25.64	100.00(66.67; 100.00)	192	55.81±30.62	66.67(33.33; 66.67)	43	85.19±18.19	100.00(66.67; 100.00)	45	**< .001**
Communication with professionals	94.62±15.25	100.00(100.00; 100.00)	192	92.25±20.36	100.00(100.00; 100.00)	43	93.33±13.48	100.00(100.00; 100.00)	45	0.510
Insecurity with the partner	55.90±27.07	66.67(33.33; 66.67)	192	42.63±28.48	33.33(33.33; 66.67)	43	62.22±28.96	66.67(33.33; 66.67)	45	**0.003**
Confidence erection	49.52±23.22	33.33(33.33; 66.67)	70	38.88±27.83	33.33(33.33; 41.66)	12	60.0±21.08	66.67(41.66; 66.67)	10	0.098
Body image (male)	64.76±30.50	66.67(33.33; 100.00)	70	50.00±38.93	50.00(25.00; 75.00)	12	86.67±23.31	100.00(75.00; 100.00)	8	**0.032**
Body image (female)	57.38±31.56	66.67(33.33; 66.67)	122	50.54±29.65	66.67(33.33; 66.67)	31	73.33±23.98	66.67(66.67;100.00)	35	**0.006**
Vaginal dryness	52.78±26.25	66.67(33.33; 66.67)	60	38.10±31.05	33.33(00.00; 66.67)	28	56.41±28.50	66.67(33.33; 66.67)	13	0.086

*Kruskal–Wallis test

Table S5. Differences in the QLQ-SH22 scales by intention of treatment

Dimensions	Curative			Palliative			P*
	M±SD	Median (interquartile range)	N	M±SD	Median (interquartile range)	N	
Sexual satisfaction	57.88±17.55	58.33(46.67; 70.83)	180	61.61±17.69	60.00(53.33; 73.33)	100	0.146
Sexual pain	40.92±26.68	33.33(30.55; 55.56)	152	40.28±29.34	33.33(16.67; 55.56)	88	0.664
Importance of sexual activity	37.41±31.64	33.33(0.00; 66.67)	152	43.33±33.00	33.33(33.33; 66.67)	88	0.134
Decreased libido	67.22±28.51	66.67(33.33; 100.00)	152	75.33±28.67	66.67(66.67; 100.00)	88	**0.012**
Worry incontinence	10.92±19.56	0.00(0.00; 33.33)	152	13.00±20.04	0.00(0.00; 33.33)	88	0.336
Fatigue	71.11±29.35	66.67(33.33; 100.00)	152	87.67±22.05	100.00(66.67; 100.00)	88	**< .001**
Treatment effect on sexual activity	72.41±28.15	66.67(66.67; 100.00)	152	81.67±23.39	100.00(66.67; 100.00)	88	**0.008**
Communication with professionals	94.81±16.08	100.00(100.00; 100.00)	152	92.67±15.41	100.00(100.00; 100.00)	88	0.071
Insecurity with the partner	53.70±26.92	66.67(33.33; 66.67)	152	57.00±30.08	66.67(33.33; 66.67)	88	0.226
Confidence erection	48.84±25.56	33.33(33.33; 66.67)	43	49.66±22.69	33.33(33.33; 66.67)	49	0.956
Body image (male)	55.81±30.62	66.67(33.33; 66.67)	43	73.47±31.17	66.67(66.67; 100.00)	49	**0.005**
Body image (female)	55.96±31.29	66.67(33.33; 66.67)	137	67.97±27.46	66.67(66.67; 100.00)	51	**0.019**
Vaginal dryness	47.62±27.52	66.67(33.33; 66.67)	84	56.86±32.84	66.67(33.33; 66.67)	17	0.269

* Mann–Whitney U test

Table S6. Differences in the QLQ-SH22 scales by ECOG performance status

Dimensions	Higher performance			Lower performance			P*
	M±SD	Median (interquartile range)	N	M±SD	Median (interquartile range)	N	
Sexual satisfaction	55.24±17.97	54.17(45.83; 66.67)	129	62.60±16.72	60.00(53.33; 73.33)	151	**0.002**
Sexual pain	39.88±27.90	33.33(22.22 ;55.56)	129	41.39±27.43	33.33(22.22 ;55.56)	151	0.580
Importance of sexual activity	36.95±30.69	33.33(,00 ;66.67)	129	41.72±33.38	33.33(33.33 ;66.67)	151	0.306
Decreased libido	64.86±28.96	66.67(33.33 ;100.00)	129	74.61±27.94	66.67(66.67 ;100.00)	151	**0.003**
Worry incontinence	9.04±17.05	0.00(0.00; 0.00)	129	13.91±21.55	0.00(0.00 ;33.33)	151	0.051
Fatigue	67.44±31.31	66.67(33.33 ;100.00)	129	85.21±21.99	100.00(66.67 ;100.00)	151	**< .001**
Treatment effect on sexual activity	70.54±28.76	66.67(33.33 ;100.00)	129	80.13±24.40	100.00 (66.67 ;100.00)	151	**0.005**
Communication with professionals	94.32±15.66	100.00(100.00 ;100.00)	129	93.82±16.06	100.00(100.00 ;100.00)	151	0.763
Insecurity with the partner	54.00±29.52	66.67(33.33; 66.67)	129	55.63±26.87	66.67(33.33; 66.67)	151	0.595
Confidence erection	46.67±24.52	33.33(33.33; 66.67)	35	50.88±23.66	33.33(33.33; 66.67)	57	0.606
Body image (male)	55.24±32.28	66.67(33.33; 66.67)	35	71.34±30.50	66.67(66.67; 100.00)	57	**0.018**
Body image (female)	56.38±31.69	66.67(33.33; 66.67)	94	62.05±29.57	66.67(33.33; 91.67)	94	0.232
Vaginal dryness	44.26±29.64	33.33(33.33; 66.67)	61	56.67±25.26	66.67(33.33; 66.67)	40	**0.045**

* Mann–Whitney U test
